# Prevalence and Genetic Identification of Three *Entamoeba* Species in Pigs in Southeastern China

**DOI:** 10.1155/2019/2824017

**Published:** 2019-11-07

**Authors:** Ting Ji, Hao-Xuan Cao, Ran Wu, Lin-Lin Cui, Guo-Ming Su, Chang Niu, Ning Zhang, Shou-Kun Wang, Dong-Hui Zhou

**Affiliations:** Key Laboratory of Fujian-Taiwan Animal Pathogen Biology, College of Animal Sciences (College of Bee Science), Fujian Agriculture and Forestry University, Fuzhou, Fujian 350002, China

## Abstract

Parasitic *Entamoeba* spp. can infect many classes of vertebrates including humans and pigs. *Entamoeba suis* and zoonotic *Entamoeba polecki* have been identified in pigs, and swine are implicated as potential reservoirs for *Entamoeba histolytica*. However, the prevalence of *Entamoeba* spp. in pigs in southeastern China has not been reported. In this study, 668 fecal samples collected from 6 different regions in Fujian Province, southeastern China, were analyzed to identify three *Entamoeba* species by nested PCR and sequencing analysis. The overall prevalence of *Entamoeba* spp. was 55.4% (370/668; 95% CI 51.6% to 59.2%), and the infection rate of *E. polecki* ST1 was the highest (302/668; 45.2%, 95% CI 41.4% to 49.0%), followed by *E. polecki* ST3 (228/668; 34.1%, 95% CI 30.5% to 37.7%) and *E. suis* (87/668; 13.0%, 95% CI 10.5% to 15.6%). *E. histolytica* was not detected in any samples. Moreover, the coinfection rate of *E. polecki* ST1 and ST3 was 25.1% (168/668; 95% CI 21.9% to 28.4%), the coinfection rate of *E. polecki* ST1 and *E. suis* was 3.7% (25/668; 95% CI 2.3% to 5.2%), the coinfection rate of *E. polecki* ST3 and *E. suis* was 0.3% (2/668), and the coinfection rate of *E. polecki* ST1, *E. polecki* ST3, and *E. suis* was 4.0% (27/668; 95% CI 2.5% to 5.5%). A representative sequence (MK347346) was identical to the sequence of *E. suis* (DQ286372). Two subtype-specific sequences (MK357717 and MK347347) were almost identical to the sequences of *E. polecki* ST1 (FR686383) and ST3 (AJ566411), respectively. This is the first study to survey the occurrence and to conduct molecular identification of three *Entamoeba* species in southeastern China. This is the first report regarding mixed infections with *E. suis*, *E. polecki* ST1, and *E. polecki* ST3 in China. More research studies are needed to better understand the transmission and zoonotic potential of *Entamoeba* spp.

## 1. Introduction

The genus *Entamoeba* comprises many free-living and parasitic species and can infect all classes of vertebrates and some invertebrates. Some *Entamoeba* species (e.g., *E. histolytica*, *E. dispar*, *E. coli*, *E. moshkovskii*, *E. hartmanni*, and *E. polecki*) have been identified in humans [1–4], and most are considered harmless, but some of these species still cause disease. Amoebiasis caused by *E. histolytica* is the third leading parasitic disease causing morbidity and mortality in humans, causing up to 50, 000 deaths per year, just behind malaria and schistosomiasis [4–7]. The disease is characterized as amebic colitis and liver abscess in humans and animals [3, 8]. Although *E. histolytica* has not been detected in farmed pigs thus far, and the susceptibility of swine to *E. histolytica* infection was revealed only under experimental conditions, swine have been considered as potential reservoirs for *E. histolytica* [9–12].

Two species, *E. suis* [13] and *E. polecki* [14], have been identified in pigs. *E. suis* appears to be mostly restricted to pigs [2, 3, 15, 16] and has been suggested to cause hemorrhagic colitis by breaking down the lamina propria [10]. Unlike *E. suis*, which infects pigs and potentially gorillas [17], *E. polecki* can infect many kinds of hosts, including humans, nonhuman primates, and pigs. The intraspecific variation of *E. polecki* was revealed by molecular analysis of the small-subunit ribosomal DNA, which showed that *E. polecki* could be divided into 4 subtypes (*E. polecki* ST1–ST4) [17, 18]. All the subtypes have been found in humans, *E. polecki* ST1 and *E. polecki* ST3 have also been found in pigs, and *E. polecki* ST2 also exists in nonhuman primates [12, 17], while human cases of *E. polecki* primarily involve *E. polecki* ST4 [19]. For a long time, *E. polecki* ST4 was only known from humans. Recently, however, ST4 was found in wild Celebes crested macaques (*Macaca nigra*) [20]. Although *E. polecki* is considered less pathogenic to humans or animals in the case of solo infection, coinfections with other pathogens, such as *Lawsonia intracellularis*, may increase the severity of the disease [3].

Swine husbandry plays an indispensable role in the animal husbandry in China. Because of the prosperity of swine husbandry and the high population density in China, the risk of exposure to zoonotic *Entamoeba* spp. is inevitable. However, the molecular epidemiology of *Entamoeba* spp. in pigs in southeastern China has not been reported. This study determined the prevalence of three *Entamoeba* species in pigs in southeastern China using molecular detection, determined the genetic identity of these *Entamoeba* species by phylogenetic analysis, and evaluated the zoonotic potential of *Entamoeba* spp.

## 2. Materials and Methods

### 2.1. Study Sampling

**Figure 1:**
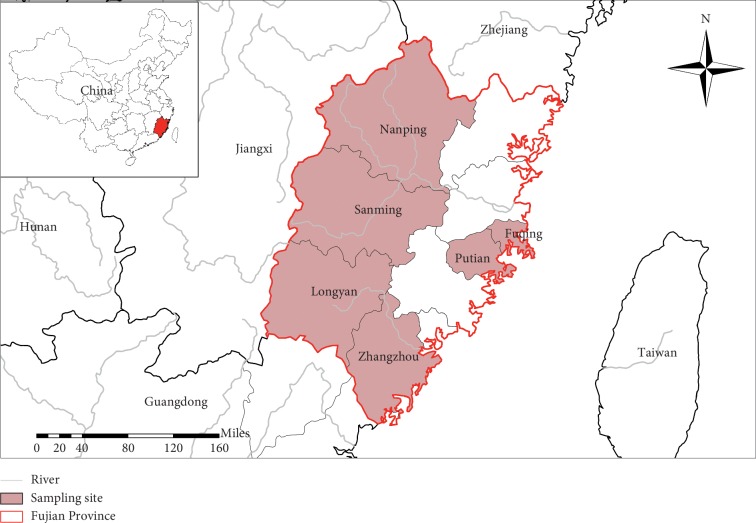
Sampling areas in Fujian Province, southeastern China.

A total of 668 fecal samples were collected from 6 regions in Fujian Province, southeastern China (Figure 1). All specimens from pigs, including weaned piglets, suckling piglets, sow, boars, nursery pigs, and fattening pigs, were collected directly from each pig's rectum or were immediately collected from the ground after defecation by the pigs. Fecal samples were marked with the corresponding sex, developmental stage, and origin of the pigs and then stored at 4°C until DNA extraction (generally within 48 hours).

### 2.2. Isolation of Genomic DNA

According to the manufacturer's instructions, genomic DNA was extracted from approximately 200 mg of each fecal samples using a Stool DNA kit (OMEGA D4015-02), and the DNA was stored at −20°C until use.

### 2.3. PCR Amplification of *Entamoeba* spp

The extracted fecal genomic DNA was used to determine the species/subtypes of *Entamoeba* spp. by nested PCR targeting the small-subunit ribosomal RNA (SSU rRNA) gene. The first set of primers, E-1 and E-2, and the second set of primers, EH-1 and EH-2, were used to detect *E. histolytica* [1]. The first round of nested PCR used primers 764–RD3, and the second round of nested PCR used primers 764–765, to identify *E. suis* [15]. The primary PCR for identifying *E. polecki* was performed using primer set Epolec F6–Epolec R6, and then the secondary PCR for subtype-specific characterization of *E. polecki* used primers Epolecki 1-Epolecki 2 (ST1) and EpST3F1-EpST3R2 (ST3) [2, 12].

An amplification reaction volume of 25 *μ*L was used to perform nested PCR. For *E. histolytica*, the reaction mixture of it contained 2.5 *μ*L DNA, 0.4 mM of each primers, 1 mM 10 × buffer (Mg^2+^ free), 0.2 mM dNTP, 1.5 mM MgCl_2_, and 0.375 U *Taq* DNA polymerase (TaKaRa, R001CM). The reaction mixture of *E. suis* contained 1 *μ*L DNA, 0.5 mM of each primers, 1 mM 10 × buffer (Mg^2+^ free), 0.2 mM dNTP, 1.5 mM MgCl_2_, and 0.625 U *Taq* DNA polymerase (TaKaRa, R001CM). The reaction mixture of *E. polecki* was similar to the reaction mixture of *E. suis*, except that each primer was used at 0.2 mM.

### 2.4. Sequencing Analysis and Phylogenetic Analysis

PCR products were separated using 1.0% agarose gels, stained with GelStain, and visualized using a UV transilluminator. The positive PCR productions were sequenced with the Big Dye Terminator v3.1 Cycle Sequencing Kit on an ABI PRISM™ 3730 XL DNA Analyzer (Applied Biosystems, Foster City, CA, USA). The accuracy of the sequences was verified with bidirectional sequencing. The obtained sequences were analyzed using the BLAST program at the NCBI website. Mega 7.0 (http://www.megasoftware.net/) software was used to perform phylogenetic analyses by the neighbor-joining method with the Kimura-2 parameter model. Bootstrap analysis with 1000 replicates was used to assess the robustness of cluster formation.

### 2.5. Data Analysis

SPSS 22.0 (IBM Corp., New York, USA) was used to analyze the data. The associations between infection rates of different sampling areas and the associations between infection rates of different developmental stages of pigs were explored using the chi-square test. Differences were considered statistically significant when *P* < 0.05.

## 3. Results

### 3.1. Prevalence of *Entamoeba* spp

A total of 370 of 668 samples (55.4%, 95% CI 51.6% to 59.2%) were positive for *Entamoeba* spp. by nested PCR (Table 1). *E. suis* and *E. polecki* were identified in fecal samples, but samples with *E. histolytica* were not found in this study. The overall infection rate of *E. polecki* ST1 was the highest (302/668, 45.2%, 95% CI 41.4% to 49.0%), while the overall infection rate of *E. suis* was the lowest (87/668; 13.0%, 95% CI 10.5% to 15.6%). The coinfection rate of *E. polecki* ST1 and *E. polecki* ST3 was the highest (168/668; 25.1%, 95% CI 21.9% to 28.4%), while the coinfection rate of *E. polecki* ST3 and *E. suis* was the lowest (2/668; 0.3%).

Analysis of the infection rates of *Entamoeba* spp. in different sampling areas showed that there were regional differences (*χ*^2^ = 167.453, *P* < 0.05), with the rates being much lower in Putian and Longyan than in other regions.

### 3.2. Distribution of *Entamoeba* spp. in Different Developmental Stages of Swine

The detailed data of distribution of *Entamoeba* spp. are shown in Table 2. Analysis of the infection rates of *Entamoeba* spp. in different developmental stages showed that there was a developmental stage predisposition to infection with *Entamoeba* spp. (*χ*^2^ = 50.362, *P* < 0.05), with the rates being much lower in suckling pigs than in other developmental stages.

### 3.3. Phylogenetic and Sequencing Analysis of *Entamoeba* spp

**Figure 2:**
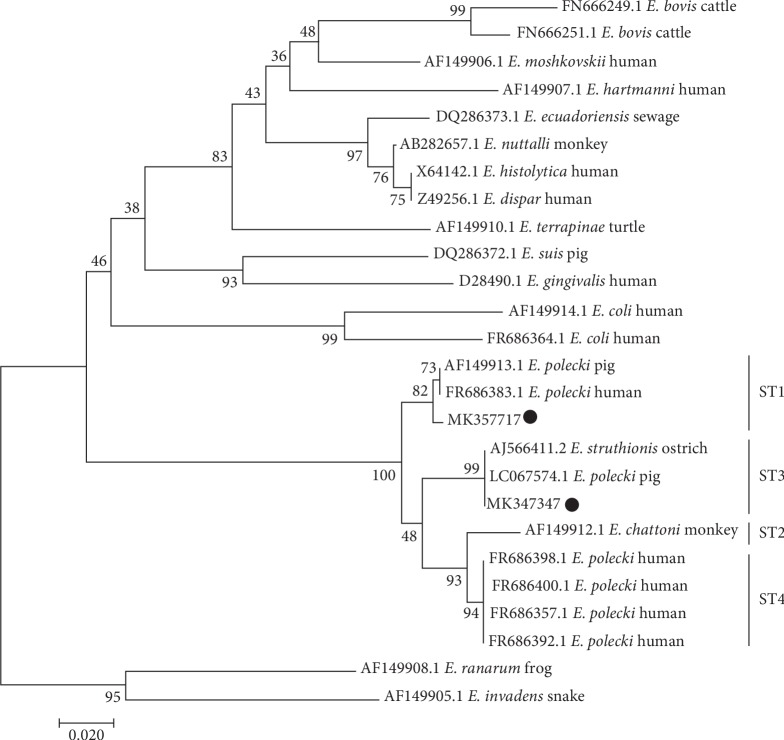
Phylogenetic relationships of *Entamoeba polecki* isolates identified in this study. The isolates obtained in this research are indicated by circles.

The positive product of *E histolytica* was not amplified by nested PCR in all samples. Representative sequences were submitted to GenBank under accession numbers MK347346 (*E. suis*), MK347347 (*E. polecki* ST3), and MK357717 (*E. polecki* ST1). Meanwhile, the three representative sequences displayed 100% sequence identity to other obtained sequences of PCR-positive samples of *E. suis* and *E. polecki* ST1 and ST3 in this study. The sequence of *E. suis* (MK347346) was identical to the sequence isolated from pigs (DQ286372). The representative sequences of *E. polecki* ST1 (MK357717) and *E. polecki* ST3 (MK347347) were almost identical to reference sequences of *E. polecki* ST1 (AF149913) and *E. polecki* ST3 (LC067574), respectively, and compared with the corresponding reference sequences, each current sequence has 1 different substitution. We chose known sequences [12] to build the phylogenetic tree of the *E. polecki* subtypes detected in the current study, and the results showed that MK357717 shared a common clade with AF149913 (*E. polecki* ST1) and MK347347 shared a common clade with LC067574 (*E. polecki* ST3) (Figure 2).

## 4. Discussion

Traditional microscopic examination is the most commonly used clinical diagnostic tool for examining the presence of *Entamoeba* organisms in fresh or fixed stool samples [1, 7, 21, 22]. However, several distinct *Entamoeba* spp. with similar morphological characteristics (for instance, the *E. dispar*, a nonpathogenic species, is morphologically identical to *E. histolytica*) cannot be distinguished by microscopic examination alone [1, 2, 4, 7]. Therefore, accurate identification of species/subtypes of *Entamoeba* was performed with molecular tools including PCR and nucleotide sequencing [1, 7, 12, 19, 23, 24].

In this study, the prevalence of *Entamoeba* spp. ranged from 21.6% to 86.4% in different regions of Fujian Province, southeastern China, and there were significant differences in the infection rates in the six areas (*P* < 0.05). The causes of these differences may be related to managing technology, breeding conditions, health status, and the water sources on farms. Moreover, the overall infection rate of *Entamoeba* spp. in this study is higher (55.4%) than the rate reported in Korea (5/136, 3.7%) [25], Iran (1/12, 8%; 2/12, 17%; 8% for *E. suis* and 17% for *E. polecki*) [26], Cambodian (24/76, 31.6%) [27], Germany (267/514, 52%) [28], and eastern China (45.8%) [12], but it is lower than that reported in Vietnam (11/12, 91.67%) [29]. These differences may be due to the different geographical variations, climates, and detection procedures.

The phylogenetic analysis indicated that the isolates from the samples for *E. polecki* were *E. polecki* ST1 and *E. polecki* ST3. Infection with *E. polecki* ST1 was the most common (45.2%) in the present study, which was consistent with the observations reported in Indonesia, Vietnam, and eastern China [12, 20, 29]. Mixed infections, including infection with *E. suis* and *E. polecki* ST1, *E. suis* and *E. polecki* ST3, *E. polecki* ST1 and *E. polecki* ST3, and *E. suis*, *E. polecki* ST1, and *E. polecki* ST3, were observed in the study. This result suggests that there is no competitive exclusion among these three species/subtypes (*E. suis*, *E. polecki* ST1, and *E. polecki* ST3). In addition, this is the first report regarding mixed infections with *E. suis* and *E. polecki* ST1 and ST3 in China. Infection with *E. histolytica* was not observed in farmed pigs in this study, which was consistent with the previous research [10–12].

Traditionally, *E. suis* was considered to be mostly restricted to pigs [2, 3, 15, 16]. However, the sequence of an *Entamoeba* isolated from a gorilla (FR686456) was similar to the sequence of *E. suis* (DQ286372) with one substitution [17], so whether *E. suis* only infects pigs should be verified by more studies. The results show that pig infection with *Entamoeba* spp. was related to the sampling areas and the developmental stages of swine (*P* < 0.05), but this is not in agreement with the observation made in pigs by Li et al. (there was no age predisposition in pigs) [12]. Therefore, more research studies are needed to confirm whether sampling area and types of swine are risk factors for *Entamoeba* spp. infection. There were only detected three *Entamoeba* species (*E. histolytica*, *E. suis*, and *E. polecki* ST1 and *E. polecki* ST3) in this study, and more research studies are needed to determine prevalence and genetic identification of other species/subtypes in pigs in China in the future.

## 5. Conclusion

The present study conducted a prevalence survey and molecular identification of three *Entamoeba* species in pigs in southeastern China. The overall infection rate of *Entamoeba* spp. was 55.4%. *E. suis* and zoonotic *E. polecki* ST1 and *E. polecki* ST3 have been found in pigs. Thus, further attention should be paid to the risk of the transmission of *Entamoeba* spp. between animal reservoirs and humans. The statistical analysis (SPSS) suggested that sampling areas and developmental stages of swine are associated with swine infection with three *Entamoeba* species. This is the first report of mixed infections with *E. suis*, *E. polecki* ST1, and *E. polecki* ST3 in China.

## Figures and Tables

**Table 1:** Occurrence of *Entamoeba* infection in pigs in Fujian Province, southeastern China.

Location (s)	Samples (*N*)	*E. histolytica*		*E. suis*		*E. polecki* ST1		*E. polecki* ST3		*E. polecki* ST1 + *E. polecki* ST3		*E. polecki* ST1 + *E. suis*		*E. polecki* ST3 + *E. suis*		*E. polecki* ST1 + *E. polecki* ST3 + *E. suis*		Total	
		*n*	Prevalence (%)	*n*	Prevalence (%)	*n*	Prevalence (%)	*n*	Prevalence (%)	*n*	Prevalence (%)	*n*	Prevalence (%)	*n*	Prevalence (%)	*n*	Prevalence (%)	*n* ^a^	Prevalence (%)
			95% CI		95% CI		95% CI		95% CI		95% CI		95% CI		95% CI		95% CI		95% CI
Sanming	118	0	0	1	0.9	80	67.8	71	60.2	60	50.8	0	0	0	0	0	0	92	78.0
			NA		NA		59.2–76.4		51.2–69.1		41.7–60.0		NA		NA		NA		70.4–85.6
Zhangzhou	81	0	0	15	18.5	69	85.2	36	44.4	35	43.2	7	8.6	0	0	8	9.9	70	86.4
			NA		9.9–27.2		77.3–93.1		33.4–55.5		32.2–54.2		2.4–14.9		NA		3.2–16.5		78.8–94.0
Nanping	120	0	0	32	26.7	74	61.7	36	30.0	31	25.8	17	14.2	1	0.8	10	8.3	83	69.2
			NA		18.6–34.7		52.8–70.5		21.7–38.3		17.9–33.8		7.8–20.5		NA		3.3–13.4		60.8–77.5
Fuqing	121	0	0	9	7.4	67	55.4	64	52.9	57	47.1	1	0.8	1	0.8	7	5.8	74	61.2
			NA		2.7–12.2		46.4–64.4		43.9–61.9		38.1–56.1		NA		NA		1.6–10.0		52.3–70.0
Putian	139	0	0	16	11.5	5	3.6	14	10.1	5	3.6	0	0	0	0	0	0	30	21.6
			NA		6.1–16.9		0.5–6.7		5.0–15.1		0.5–6.7		NA		NA		NA		14.7–28.5
Longyan	89	0	0	14	15.7	7	7.9	7	7.9	5	5.6	0	0	0	0	2	2.3	21	23.6
			NA		8.0–23.4		2.2–13.6		2.2–13.6		0.7–10.5		NA		NA		NA		14.6–32.6
Total	668	0	0	87	13.0	302	45.2	228	34.1	168	25.1	25	3.7	2	0.3	27	4.0	370	55.4
			NA		10.5–15.6		41.4–49.0		30.5–37.7		21.9–28.4		2.3–5.2		NA		2.5–5.5		51.6–59.2

*N*: number of samples examined; *n*: number of positive samples; ^a^including multiple infections; 95% CI: 95% confidence interval; NA: not applicable.

**Table 2:** *Entamoeba* spp. detected among different pig developmental stage groups in Fujian Province, southeastern China.

Growing stage (s)	Samples (*N*)	*E. histolytica*		*E. suis*		*E. polecki* ST1		*E. polecki* ST3		*E. polecki* ST1 + *E. polecki* ST3		*E. polecki* ST1 + *E. suis*		*E. polecki* ST3 + *E. suis*		*E. polecki* ST1 + *E. polecki* ST3 + *E. suis*		Total	
		*n*	Prevalence (%)	*n*	Prevalence (%)	*n*	Prevalence (%)	*n*	Prevalence (%)	*n*	Prevalence (%)	*n*	Prevalence (%)	*n*	Prevalence (%)	*n*	Prevalence (%)	*n* ^a^	Prevalence (%)
			95% CI		95% CI		95% CI		95% CI		95% CI		95% CI		95% CI		95% CI		95% CI
Weaned piglet	116	0	0	2	1.7	64	55.2	64	55.2	45	38.8	0	0	0	0	2	1.7	81	69.8
			NA		NA		46.0–64.4		46.0–64.4		29.8–47.8		NA		NA		NA		61.3–78.3
Sucking piglet	105	0	0	3	2.9	21	20.0	12	11.4	7	6.7	1	1.0	1	1.0	0	0	27	25.7
			NA		NA		12.2–27.8		5.2–17.6		1.8–11.5		NA		NA		NA		17.2–34.2
Sow	280	0	0	62	22.1	139	49.6	86	30.7	64	22.9	21	7.5	1	0.4	18	6.4	167	59.6
			NA		17.2–27.0		43.8–55.5		25.3–36.2		17.9–27.8		4.4–10.6		NA		3.5–9.3		53.9–65.4
Boar	28		0	0	0	13	46.4	12	42.9	11	39.3	0	0	0	0	0	0	14	50.0
			NA		NA		26.7–66.1		23.3–62.4		20.0–58.6		NA		NA		NA		30.3–69.7
Nursery pig	90	0	0	12	13.3	45	50.0	42	46.7	29	32.2	2	2.2	0	0	7	7.8	54	60.0
			NA		6.2–20.5		39.5–60.5		36.2–57.2		22.4–42.1		NA		NA		2.1–13.4		49.7–70.3
Fattening pig	49	0	0	8	16.3	20	40.8	12	24.5	12	24.5	1	2.0	0	0	0	0	27	55.1
			NA		5.6–27.1		26.6–55.1		12.0–37.0		12.0–37.0		NA		NA		NA		40.7–69.5
Total	668	0	0	87	13.0	302	45.2	228	34.1	168	25.1	25	3.7	2	0.3	27	4.0	370	55.4
			NA		10.5–15.6		41.4–49.0		30.5–37.7		21.9–28.4		2.3–5.2		NA		2.5–5.5		51.6–59.2

*N*: number of samples examined; n: number of positive samples; ^a^including multiple infections; 95% CI: 95% confidence interval; NA: not applicable.

## Data Availability

The data used to support the findings of this study are included within the article.
